# Inter-element orientation and distance influence the duration of persistent contour integration

**DOI:** 10.3389/fpsyg.2014.01273

**Published:** 2014-11-06

**Authors:** Lars Strother, Danila Alferov

**Affiliations:** ^1^Brain and Mind Institute, University of Western OntarioLondon, ON, Canada; ^2^Cognitive and Brain Sciences Program, Department of Psychology, University of Nevada RenoReno, NV, USA

**Keywords:** contour integration, form perception, perceptual organization, perceptual grouping, association field, collinearity, perceptual hysteresis, visual persistence

## Abstract

Contour integration is a fundamental form of perceptual organization. We introduce a new method of studying the mechanisms responsible for contour integration. This method capitalizes on the perceptual persistence of contours under conditions of impending camouflage. Observers viewed arrays of randomly arranged line segments upon which circular contours comprised of similar line segments were superimposed via abrupt onset. Crucially, these contours remained visible for up to a few seconds following onset, but eventually disappeared due to the camouflaging effects of surrounding background line segments. Our main finding was that the duration of contour visibility depended on the distance and degree of co-alignment between adjacent contour segments such that relatively dense smooth contours persisted longest. The stimulus-related effects reported here parallel similar results from contour detection studies, and complement previous reported top–down influences on contour persistence (Strother et al., 2011). We propose that persistent contour visibility reflects the sustained activity of recurrent processing loops within and between visual cortical areas involved in contour integration and other important stages of visual object recognition.

## INTRODUCTION

The perceptual binding of spatially local edge information into global contours, or *contour integration*, is a crucial stage of visual object recognition. Contour integration is subject to both bottom–up and top–down influences that depend on stimulus regularities, expectations, task demands, and other factors (Hess and Field, 1999; Gilbert and Li, 2013). Much of the psychophysical work on contour integration in human vision involves measuring the detectability of contours embedded in highly camouflage backgrounds. Here we introduce a new method of studying contour integration and its underlying mechanisms. This method is complementary to traditional contour detection methods, and relies on the perceptual decay of a contour under conditions of impending camouflage.

Regan (1986) noted that a highly camouflaged shape (e.g., the outline of a bird) made visible by motion does not disappear immediately after it stops moving^1^. Indeed, several studies of this phenomenon have since shown that outlines of recognizable objects and simple shapes persist perceptually for up to several seconds, and furthermore, this persistence of global form is accompanied by persistent neural activity in V1 and higher-tier visual cortical areas (Ferber et al., 2003; Large et al., 2005; Strother et al., 2011, 2012). These findings demonstrate a unique type of perceptual hysteresis, which we refer to here as *contour persistence*. Contour persistence is distinct from other varieties of visual persistence, both in terms of the stimulus conditions under which it occurs and also its duration. Contour persistence occurs following the offset of a perceptual segmentation cue (e.g., onset or motion) rather than the physical removal of the contour itself or any of the elements comprising the contour. This makes contour persistence distinct from “visible persistence” phenomena (Coltheart, 1980a,b) in which a stimulus continues to be perceived following its physical offset. Contour persistence also differs from other types of visual persistence in terms of its relatively long duration—contour persistence typically lasts >1 s, whereas other visual persistence phenomena typically last <1 s.

Here we measured the duration of contour persistence using a contour fading paradigm in which a circular contour comprised of line segments abruptly onset against a background of randomly oriented line segments^2^. We found that such contours did not disappear immediately following onset, but instead became camouflaged over the course of a few seconds, as in the earlier demonstration (Regan’s bird). Our main goal was to measure the duration of contour persistence as a function of known determinants of contour binding strength (contour smoothness, density, and closure). Complementary to psychophysical studies of contour integration (e.g., Field et al., 1993; Pettet, 1999; Bex et al., 2001; Ledgeway et al., 2005; May and Hess, 2007, 2008; Dakin and Baruch, 2009; Marotti et al., 2012), we used contours comprised of elements that were either co-aligned and tangent to the contour (*snake* contours), co-radial (co-parallel and perpendicular to the contour; *ladder* contours), or randomly oriented (*jagged* contours). The first question of interest in our study was whether or not local orientation influences the duration of contour persistence. There is substantial evidence of an *association field* (Field et al., 1993) mechanism in visual cortex that enables contour detection of visual elements (e.g., line segments, wavelets) camouflaged within an array of similar elements. The association field consists of neural units tuned to specific orientation, which facilitate the activity of other neural units tuned to similar orientations but at different locations within the visual field, and thus facilitate contour binding and detection. We wondered whether or not local orientation might play a similar role in persistent contour integration. If so, it is possible that the association field maintains a persistent representation of a contour, and thus exhibits visual memory (Magnussen, 2000), either due to the reverberation of feedforward and feedback signals within the neural association field itself, or by virtue of feedback from higher tier visual cortical areas.

We performed additional experiments to examine the effects of relative density and closure contour persistence, both of which have been studied using detection paradigms (Smits et al., 1985; Kovács and Julesz, 1993; Tversky et al., 2004; Mathes and Fahle, 2007), but have not previously been manipulated in studies of contour persistence. We reasoned that if more strongly bound contours persisted longer than less strongly bound contours, this would demonstrate a stimulus-driven influence on the duration of contour persistence. Finally, we performed a control experiment to determine whether or not our results could be accounted for by eye movements. We discuss our results in terms of a recurrent process of feedforward contour integration in primary visual cortex (V1) and shape-related feedback from higher-tier visual cortical areas.

## MATERIALS AND METHODS

### SUBJECTS

All observers had normal or corrected-to-normal vision. Ten observers participated in Experiment 1. Six new observers participated in Experiment 2 and Experiment 3a. Two new observers (and one observer from the previous experiments) participated in a final control experiment (Experiment 3b) which employed eye-tracking. All observers provided informed consent and were recruited in accordance to University of Western Ontario ethics guidelines.

### STIMULI AND PROCEDURE

**FIGURE 1:**
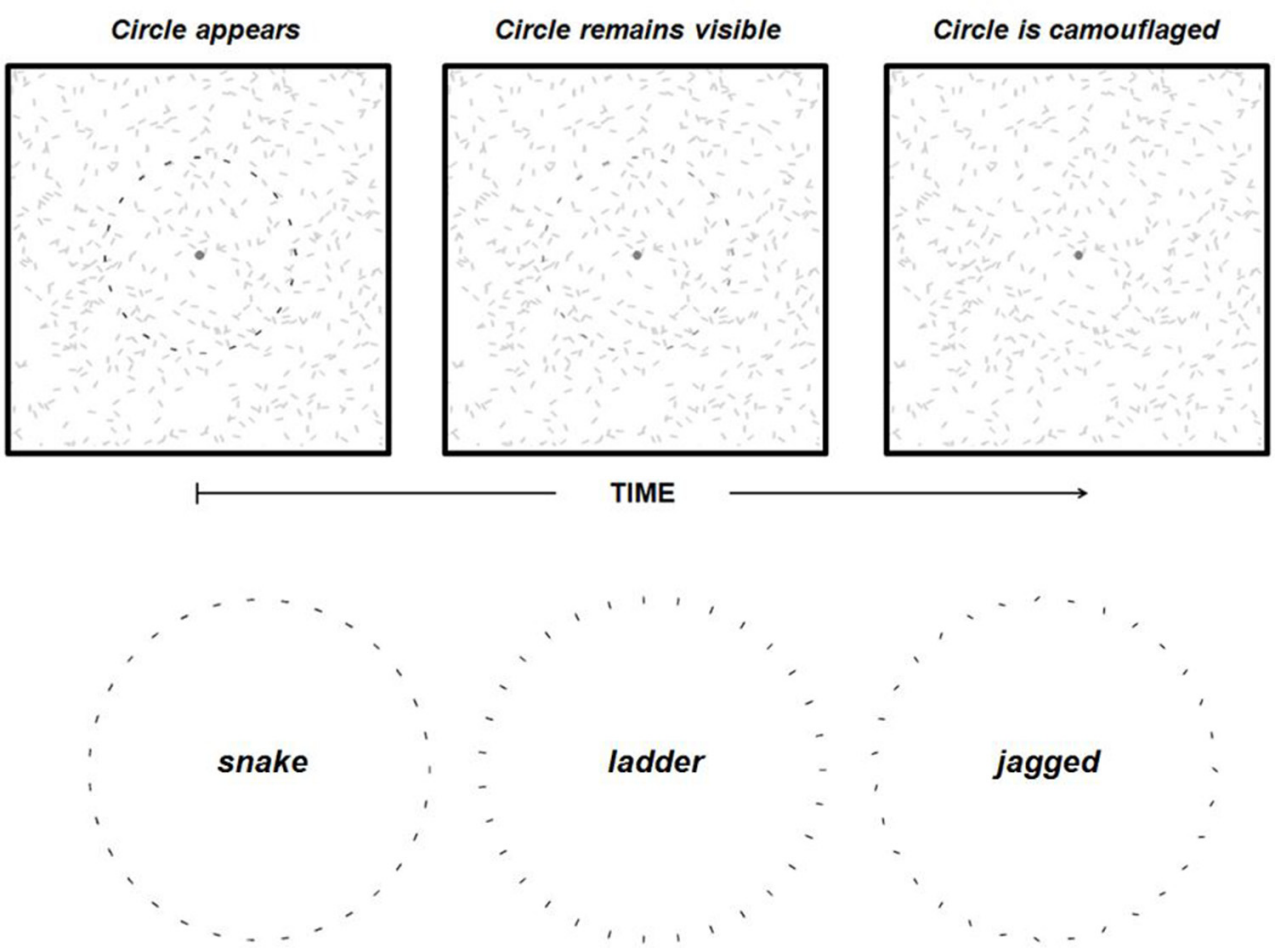
**The top three panels illustrate the contour fading paradigm.** When a circle comprised of discrete elements appears (top left panel) against a background of similar elements it remains visible (top middle panel) for up to several seconds but eventually becomes camouflaged (top right panel), and it is perceived to have disappeared even though the circle is still physically present. Note that the circle is darkened in the left two panels to illustrate its perceptibility rather than an actual difference in luminance between circle elements and background elements. The three inter-element alignment conditions used in Experiment 1 are also shown (bottom).

All experiments employed stimuli comprised of short line segments (**Figure 1**^2^). Trials began with the appearance of a ‘background’ array of randomly oriented dark line segments (∼0.3° × 0.03°) positioned randomly (overlap allowed) within a lighter 10° × 10° square aperture on an otherwise dark display. A blue fixation cross (∼0.3° × 0.3°) was always present in the center of the aperture during the experiments. Shortly (2 s) after the appearance of the background array, a circle or semi-circle comprised of line segments identical to those comprising the background appeared against the background and remained until the end of the trial (total trial duration was always 8 s). We used *snake* circles comprised of co-circular elements (i.e., smooth contours), *ladder* circles comprised of co-radial (rotated 90° from co-circular), and *jagged* circles comprised of randomly oriented elements (random orientations were generated trial to trial). The absolute positions of each of the elements along a circle or semi-circle were equivalent across all three stimulus types and conditions.

Observers were instructed to maintain fixation throughout the experiment and, on each trial, to press a button when the circle (or semi-circle) was no longer visible; this served as a measure of response time (RT). At the end of each trial a new background array appeared and the sequence was repeated. The appearance of a new background on each trial completely replaced that of the previous trial (novel background elements were generated on every trial). Individual trials ended either with the button press or after 6 s if no button was pressed. Observers were told not to press the button if they never saw the target or if any portion of it never became fully camouflaged (i.e., did not disappear), which occurred on less than 2% of trials for all observers. Observers were always given at least 25 practice trials, the results of which were not included in our analyses. Individual observers completed at least 100 trials in each experiment. Pilot studies for each observer confirmed that circular contours that disappeared were never detectable were it not for the onset cue (i.e., observers could not see the contours when the contour was superimposed against the background in the absence of an onset cue).

**FIGURE 2:**
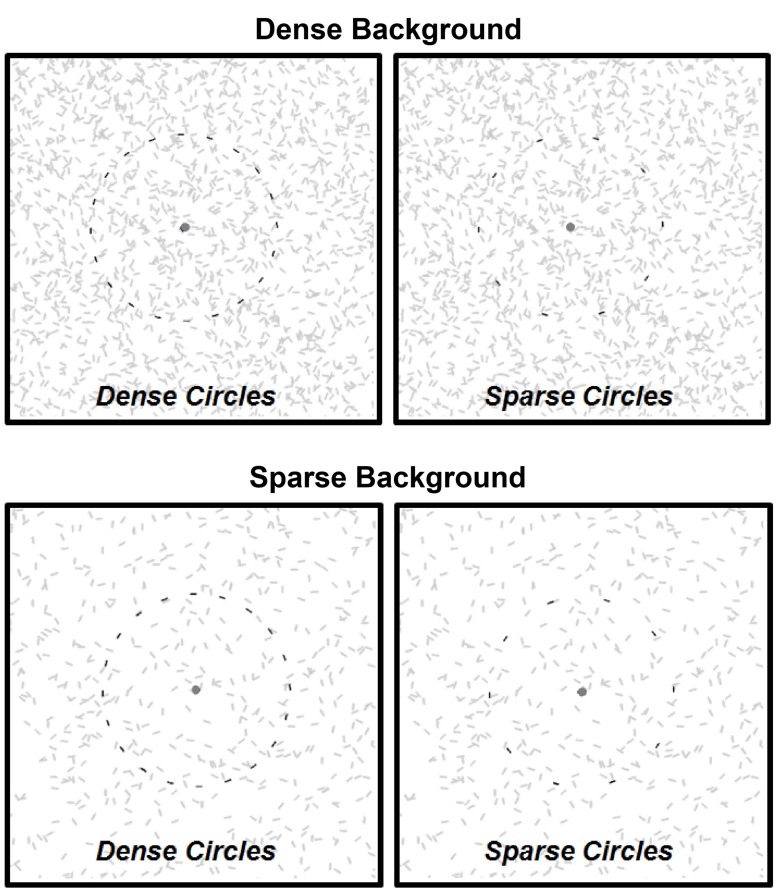
**Circles of different three densities appeared against backgrounds of three different densities; two of extremes for each shown here.** Non-smooth circles were identical to those shown except that the orientation of each element was random. The circles shown here are darkened for purpose of illustration only; all line segments were of equal luminance during the experiment (see Materials and Methods).

In Experiment 1 we were primarily interested in whether or not the duration of continued contour visibility depended on inter-element alignment: *smooth* (“snake”) co*-radial* (“ladder”) or *jagged* circles. We also varied the size of the circles and the proximity of the elements making up each circle (*circle density*), and also the density of the background elements (*background density*). We used three circle densities: line segments covered ∼33, 25, or 20% of a given circle’s circumference. Backgrounds consisted of 2250, 3000, or 3750 elements per 10° × 10° area. Examples of maximally dense or sparse contour-background pairings are shown in **Figure 2** (contour and background elements are shown in different colors to make the contour elements visible; all were the same color in the experiments).

The purpose of varying circle and background density in this experiment was mainly to reduce the predictability of the location of the circle elements (we explore these variables further in Experiment 2). By increasing the variability in the density of the circles relative to that of the background we hoped to increase the overall variability in the latencies of individuals’ button presses. Individual observers thus completed at least 33 trials per alignment condition.

In Experiment 2 we further investigated prospective effects of circle and background densities and circle size using *smooth* (snake) circles and *jagged* circles. We were particularly interested in comparing the magnitude of durations for these two alignment conditions to that of the relative densities of the circles and backgrounds. We again used three circles sizes; for each circle size (radii of 1.5, 3, and 4.5°), *dense* circles were comprised of 10, 20, and 30 line segments (respectively), and for *sparse* circles the number of line segments was halved. We also used sparse (500 elements) and dense (1500 elements) backgrounds (per 10° × 10° area, as in Experiment 1); the condition pairings are illustrated in **Figure 2**.

In Experiment 3a we tested whether or not the effects observed in Experiment 2 could be observed for non-closed contours (semi-circles created from 0.5 × the circumference of the circles used in Experiment 2) by re-testing the same subjects (from Experiment 2) and manipulating a subset of the parameters used in Experiment 2. The location of the arcs in Experiment 3a varied between the upper and lower visual hemifield, and ±2° from fixation (thus resulting in greater position uncertainty than in the previous experiments). We ran fewer observers in Experiments 2 and 3a than we did in Experiment 1, but we collected at least twice the amount of data per subject. The primary motivation for this experiment was to test whether or not closure would act as a cue above and beyond inter-element alignment. Finally, in Experiment 3b, we used an eye-tracker (Eyelink; SR Research Ltd., Toronto, ON, Canada) to monitor the eye movements of three observers in a partial replication of Experiment 2. We allowed circle size to vary from 2.7 to 4.5° visual angle; the circles were either smooth (snakes) or jagged (100 trials of each condition), and relative density was held constant (sparse circles on sparse background, as described earlier in this section).

## RESULTS

### EXPERIMENT 1

**FIGURE 3:**
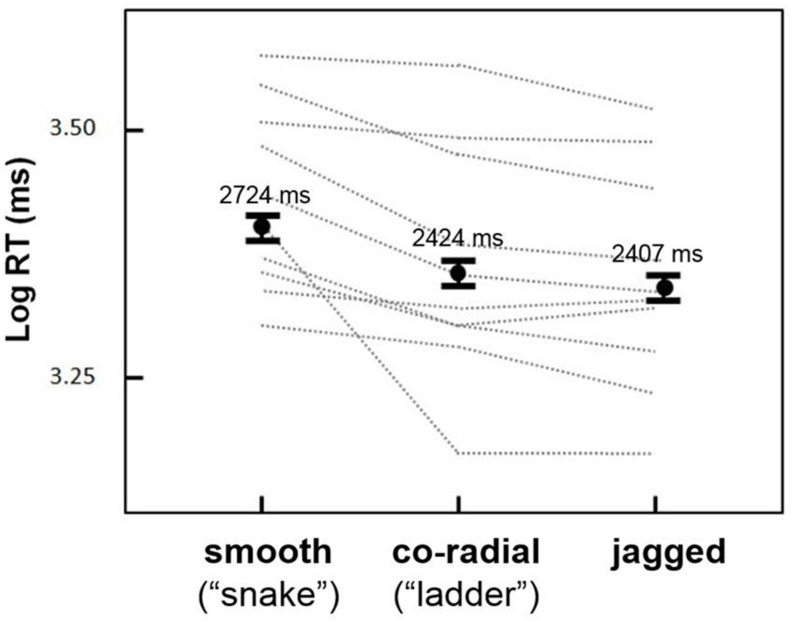
**Results from Experiment 1.** Mean log RTs (black dots) for the three inter-element alignment conditions. *Snake* circles remained visible the longest, followed by *ladder* and *jagged* circles (error bars are 95% confidence intervals). Gray dashed lines show results for individual observers, all of whom showed a similar trend for *snake* versus *ladder* and *jagged* contours; not all subjects showed *ladder* > *jagged* log RTs.

The goal of the first experiment was to test for an effect of inter-element alignment on the duration of persistent contour visibility. Mean RTs were greatest for *smooth* contours (2724 ms), followed by *co-radial* contours (2424 ms) and *jagged* contours (2407 ms). For all remaining statistical analyses RTs were log-transformed to reduce positive skew. Log RTs for the three alignment types are shown in **Figure 3**.

Preliminary repeated measures analyses of variance (ANOVAs) showed no significant main effects or interactions of circle size, circle density, or background density (possibly due to the low number of trials for each condition; we explore these variables further in the next experiment) on log RT, although the interaction of alignment type and circle density approached statistical significance [*F*(1,9) = 3.5, *p* = 0.09]. A subsequent repeated measures ANOVA based on the three alignment conditions (*smooth*, *c-radial*, *jagged*) showed a highly significant effect of alignment [*F*(1,9) = 13.9, *p* < 0.005]. *Post hoc* analyses (paired samples *t*-tests, one-tailed) showed significant differences between all three conditions: *smooth* > *co-radial* [*t*(9) = 3.38, *p* < 0.01]; *smooth* > *jagged* [*t*(9) = 3.73, *p* < 0.01]; *co-radial* > *jagged* [*t*(9) = 2.06, *p* < .05]. The *smooth* contours thus evinced a ∼300 ms increase in RT relative to *co-radial* and *jagged* contours. Although the difference between *co-radial* and *jagged* contours was also statistically significant (*co-radial* > *jagged*), this difference was relatively small (∼15 ms) compared to the *smooth* > *co-radial* and *smooth* > *jagged* differences, and less consistent across subjects (**Figure 3**; two subjects showed either no difference or greater RTs for *jagged* versus *so-radial* contours).

### EXPERIMENT 2

**FIGURE 4:**
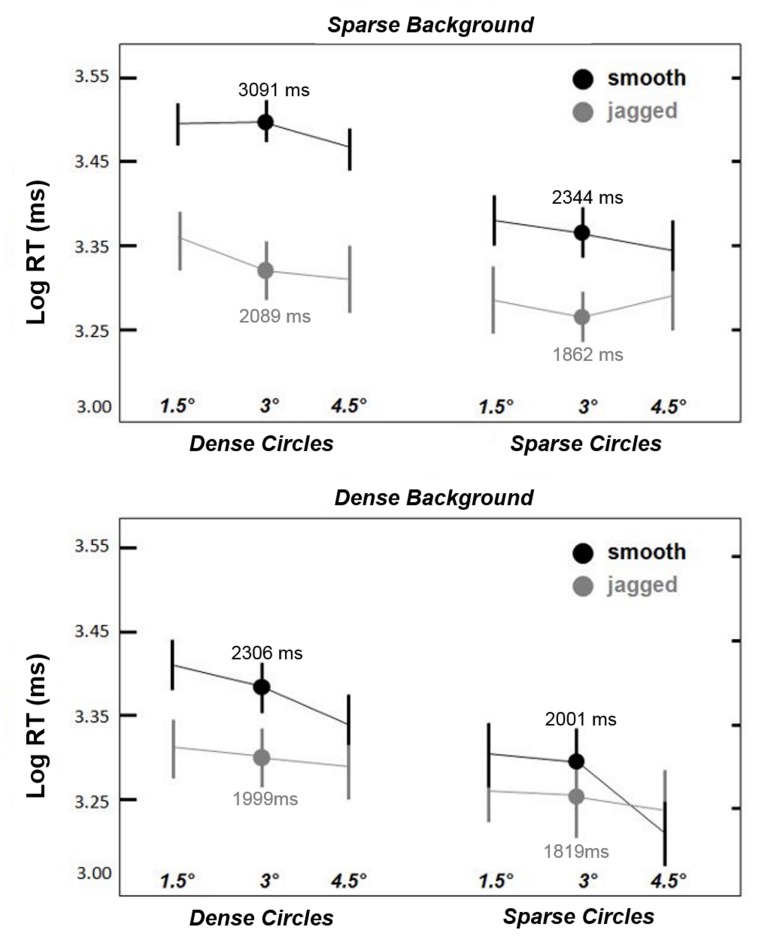
**Results from Experiment 2.** As in Experiment 1, smooth *snake* contours always remained visible longer than *jagged* contours. This effect was observed to be greatest for dense circles, and weakest for sparse circles paired with relatively dense backgrounds (lower right). The three circles sizes are indicated in ° visual angle along the x-axis. Dots indicate mean log RT for the intermediate circle size (3°). Error bars are 95% confidence intervals.

As in Experiment 1, all analyses were conducted on log RTs. **Figure 4** shows that *smooth* circles remained visible longer than *jagged* circles, and the *smooth* > *jagged* log RT trend is apparent across all pairings of circle size, circle density, and background density (except possibly for *sparse* circles paired with *dense* backgrounds, shown in the lower right of **Figure 4**). A repeated measures ANOVA yielded statistically significant main effects for all factors except circle size: *alignment* [*F*(1,5) = 13.2, *p* < 0.05]; *circle density* [*F*(1,5) = 18.4, *p* < 0.01]; and *background density* [*F*(1,5) = 22.2, *p* < 0.01]; although there appears to be a trend in **Figure 4** of decreased log RT with increasing circle size (for *smooth* circles), this was not significant [*F*(1,5) = 2.0, *p* = 0.19], which means that the effect of alignment was largely scale invariant within 4.5° from fixation.

Two-way interactions between alignment and density were also statistically significant: *alignment* × *circle density* [*F*(1,5) = 11.7, *p* < 0.05]; and *alignment* × *background density* [*F*(1,5) = 9.5, *p* < 0.05]. These interactions are apparent in **Figure 4** in that the effect of alignment (*smooth* > *jagged*) on log RT was greatest for dense circles and sparse backgrounds. No significant density interaction (*circle density* × *background density*) was observed [*F*(1,5) = 0.1, *p* = 0.73]. A three-way interaction between these variables (*alignment*× *circle density* × *background density*) was also significant [*F*(1,5) = 18.2, *p* < 0.01]. Paired-samples *t*-tests confirmed that *smooth* > *jagged* log RTs across all combinations of circle and background density [*t*(5) = 2.8–4.1, always *p* < 0.05, two-tailed], except for *sparse* circles and *dense* backgrounds [*t*(5) = 2.3, *p* = 0.07], which approached statistical significance. Thus, while effect of alignment (*smooth* versus *jagged*) varied with the relative density of the circle and background elements, *jagged* circles always tended to disappear more quickly than *smooth* circles. In short, the results shown in **Figure 4** indicate the greatest log RTs for dense *smooth* circles superimposed on sparse backgrounds.

### EXPERIMENT 3a

**FIGURE 5:**
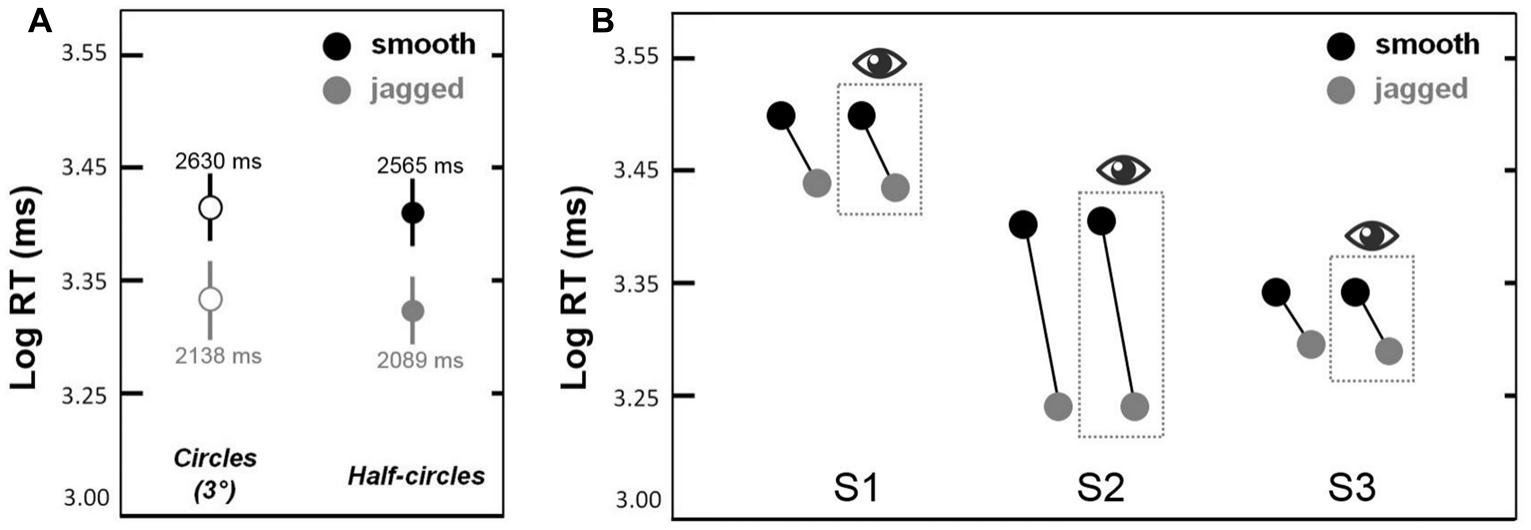
**Results from Experiments 3a and 3b.** In **(A)**, contour persistence (mean log RT) was greater for smooth circles and semi-circles as compared to jagged circles and semi-circles. Error bars are 95% confidence intervals. In **(B)**, contour persistence was greater for smooth versus jagged contours, even when trials with eye movements that deviated beyond 1.5° from fixation were omitted (the latter are shown in the dashed box with the eye symbol above).

This experiment was a partial replication of Experiment 2 (same observers) in which we sought to replicate the *smooth* > *jagged* log RT result for non-closed contours (semi-circles). **Figure 5A** shows mean log RTs corresponding to circles (solid bars) and semi-circles (dots with error bars) obtained in Experiment 3a. The same *smooth* > *jagged* log RT trend was observed in all three cases. A repeated measures ANOVA showed a main effect of inter-element alignment [*smooth* > *jagged*; *F*(1,5) = 7.7, *p* < 0.01]; a main effect of closure (with circles persisting longer than semi-circles) approached significance [*F*(1,5 = 2.9, *p* = 0.09], and there were no significant interactions. This means that the *smooth* > *jagged* effect shown in **Figures 3** and **4** is not limited to closed contours.

### EXPERIMENT 3b

The purpose of this experiment was to determine whether or not the greater persistence of smooth (snake) contours versus jagged contours could be explained by differences in eye movements. Our logic was as follows: if differences in eye movements explains our results, then equating for eye movements between our two conditions—*smooth* and *jagged*—should result in equivalent contour persistence durations. **Figure 5B** shows similar contour persistence durations (*smooth* duration > *jagged* duration) for the data based on all 200 trials (100 *smooth*, 100 *jagged*). For all three subjects, discarding trials during which gaze shifted beyond 1.5° from fixation, resulted in the exclusion of >25% of the original data. Nevertheless, as is clear in **Figure 5B**, this filtering of the original trials had no effect on the pattern of results, namely that smooth contours consistently persisted longer than jagged contours. The results in the dashed box in **Figure 5B** are based on the filtered data, and results from the original data are shown to the left of each box. The slopes of the lines connecting the black dots (smooth) and gray dots (jagged) are similar for all within-subject pairings. This means that the *smooth* > *jagged* duration result is not due to the effects of eye movements toward the circular contours used in each condition. Even when eye movements were restricted to within 1.5° from the fixation cross, and did not impinge on even the smallest circle used in the experiment (radius = 2.7°), the influence of inter-element alignment on contour persistence was the same (**Figure 5B**). Additional eye movement results reported in Supplementary Material (Figure S1).

## DISCUSSION

We used a new perceptual fading paradigm to study persistent contour integration under conditions of impending camouflage. In Experiments 1 and 2, we found that the duration of contour persistence was influenced by stimulus properties known to influence contour salience in traditional contour detection paradigms, namely inter-element distance and co-alignment (Smits et al., 1985; Field et al., 1993; Geisler et al., 2001; Elder and Goldberg, 2002). Given major differences between our contour fading paradigm and detection paradigms typically used to study contour integration, this was not an inevitable result. For instance, the contours used in our study were undetectable were it not for the onset cue, and thus synchronous onset alone could have resulted in persistence (Wong et al., 2009), without the additional influence of inter-element alignment. Furthermore, the results observed here cannot be fully accounted for by differences in eye movements for smooth versus non-smooth contours (Experiment 3b). The persistent visibility of highly camouflaged contours observed in our study is consistent with a recurrent processing loop in which high-tier neural representations global form interact with low-level neural mechanisms that bind local edges into global contours. Given the absence of contour persistence when contour elements are physically removed (Ferber et al., 2003; Wong et al., 2009; Strother et al., 2012), it is highly plausible that feedforward responses in primary visual cortex provide a neural basis upon which all feedback effects are exerted.

### CONTOUR PERSISTENCE AND THE ‘ASSOCIATION FIELD’

It has long been recognized that the sensation produced by a visual stimulus can persist after its offset, and the term “visual persistence” has been used to denote many different examples of short-term perceptual memory. Coltheart (1980a,b) used “visible persistence” to refer to cases when a visual stimulus continues to be perceived after its offset, and to distinguish these cases from “iconic memory” (Sperling, 1960), which is not accompanied by persistent perception of a physically absent stimulus. In contrast to both visible persistence and iconic memory, contour persistence occurs in the absence of physical removal (offset) of the contour. Indeed, several previous studies showed that global contours do not persist when the elements comprising the contour are removed (Ferber et al., 2003; Large et al., 2005; Wong et al., 2009; Strother et al., 2011, 2012). That is, contour persistence reflects the sustained perceptual organization of elements after an initial binding cue (onset in this case) has ended, rather than the sustained perceptual representation of a visual stimulus that has physically disappeared. Furthermore, contour persistence typically lasts considerably longer than iconic memory and other types of short-term visual memory (which are usually <1 s).

The results of the present study showed clear influences of physical properties of a contour on the duration of its perceptual persistence under camouflaging conditions. Experiment 1 showed that smooth contours showed the greatest degree of persistent contour visibility. When elements were equally co-aligned but perpendicular to the tangent of the circular global contour (the *co-radial* condition), the facilitative effect of inter-element alignment on contour persistence was reduced (**Figure 3**). Randomizing the orientations of contour elements in the *jagged* condition had a similar (but slightly greater) effect, and showed the weakest degree of persistence of the three contour conditions used in Experiment 1. The difference in duration of contour persistence for *snake* and *jagged* contours (*smooth* > *jagged*) was replicated in Experiment 2, and shown to be modulated by inter-element distance, such that decreasing the inter-element distances of the contour elements relative to the background elements decreased or eliminated the *smooth* > *jagged* effect (**Figure 4**). Experiment 3a showed that similar effects of co-alignment and density are not limited to closed circular contours (**Figure 5**), although an additional facilitative effect of closure is nevertheless a possibility—circular arcs (semi-circles) did not persist as long in general, but this trend was not statistically significant. While it is well-known that for curved contours comprised of discrete oriented elements, smooth contours are easier to detect than jagged contours, this study is the first to show that increasing contour density and smoothness facilitate contour persistence under conditions of extreme camouflage. Previous studies have recognition-related effects on the duration of visual persistence (Ferber et al., 2005; Ferber and Emrich, 2007; Emrich et al., 2008; Strother et al., 2011), but none of these systematically manipulated contour properties in a manner consistent with detection studies of contour integration. The results of the present study are thus an important step toward identifying common mechanisms involved in contour integration and contour persistence, and the relationship of these mechanisms to feedforward and feedback processes in human vision.

Hebb (1949) proposed that short-term memory consists in the persistent reverberation of activity in neuronal assemblies. A plausible explanation of the results of the present study is that contour persistence reflects persistent reverberation of an *association field* mechanism in visual cortex (Field et al., 1993). The association field is a neuronal assembly consisting of cells with similar orientation preferences and receptive fields at different retinal locations. These cells exhibit mutually facilitative interactions, and the more similar adjacent cells are in receptive field location and orientation preference, the stronger the facilitation. This mechanism thus shows greater mutual facilitation with increasing edge co-alignment. It is plausible that an association field mechanism is responsible, at least in part, for both the initial perception and persistence of global contours in the present study. This would be consistent with the effects of inter-element distance and co-alignment on the duration of persistence reported here, which parallel similar effects on the detectability of contours (e.g., Field et al., 1993; Bex et al., 2001; Ledgeway et al., 2005; Marotti et al., 2012).

### FEEDFORWARD AND FEEDBACK INFLUENCES

Our findings are consistent with the view that neural mechanisms in higher-tier visual cortical areas represent hypotheses about low-level visual input, and in doing so, reinforce inferences (e.g., about shape) via feedback to lower level visual cortical mechanisms to facilitate efficient extraction and encoding of visual features (Engel et al., 2001; Murray et al., 2002; Cardin et al., 2011). There is growing consensus that top–down feedback plays an integral role in contour integration (Gilbert and Li, 2013), but the precise nature of the effects of this feedback is not known. One possibility is that feedforward contour integration processes are accompanied by feedback processes that serve to disambiguate and enhance the salience of global contour by suppressing background noise (Strother et al., 2012; Chen et al., 2014). In this framework, extrastriate feedback could serve to modulate the responses of neurons in primary visual cortex (V1). More specifically, the responses of neurons stimulated by background elements would be suppressed and the responses of neurons stimulated by contour elements would be facilitated by extrastriate feedback in addition to facilitation by an association field within V1. The crucial result of this feedback would be the facilitation of inter-element binding within the contour and the suppression of background noise, and ultimately, the perceptual segmentation of the contour from its surroundings.

In addition to the facilitation of contour binding by an association field in V1, extrastriate feedback may also play an important role, not only in contextually modulating the responses of individual V1 neurons (Zipser et al., 1996; Lamme et al., 1998), but also in temporarily sustaining the joint activity of neurons in the association field. It is worth noting that the duration of contour persistence in the *jagged* condition (**Figure 4**) was surprisingly long, even when the density of these jagged contours was similar to that of the background. This surprising effect highlights the importance of synchronous onset in the persistence of global form (Wong et al., 2009), and role of temporal synchrony as a powerful binding cue in contour integration (Usher and Donnelly, 1998; Beaudot, 2002), and even higher stages of the visual object recognition process (Singer and Kreiman, 2014). The fact that synchronous neuronal firing is a common feature of neural network models of contour integration and other types of perceptual organization (Sporns et al., 1991; Yen and Finkel, 1998), it is conceivable that the synchronous onset of elements comprising a contour could result in the persistent activity of visual cortical neurons irrespective of contour smoothness. This prediction is consistent with findings that global contours are represented in shape-selective cortex irrespective of the local features (Altmann et al., 2003; Kourtzi et al., 2003).

Taken together with the results of previous studies (Strother et al., 2011, 2012), the results reported here lead us to propose that contour persistence reflects sustained feedforward and feedback visual processing. Some of this processing involves the binding of local visual elements into global form, which involves feedforward processing in visual cortex as well as feedback processing, both within and between visual cortical areas (Chen et al., 2014). Our results show that this complex circuit exhibits short-term memory, as evidenced by the persistence of a contour under conditions of impending camouflage. It is not clear whether the persistent contour integration reported here is due to hysteresis intrinsic to mechanisms in visual cortex alone—for example, sustained neural reverberation within the association field—or involves neural reverberation at a larger cortical scale, such as a recurrent processing loop between shape-selective neural mechanisms in extrastriate visual cortex (Kourtzi and Connor, 2011), and those in earlier visual cortical areas (e.g., V1). For instance, feedforward activity in V1 could be subsequently modulated by interactions within the association field, which may serve to enhance the perceptual salience of a contour relative to its background (Gilbert and Li, 2013). Additionally, shape-related feedback from V4 and higher-tier areas could exert an additional influence on the responses of V1 neurons, by facilitating the responses of those corresponding to contour elements, and by inhibiting the responses of neurons responding to background elements (Chen et al., 2014). Persistent contour integration could therefore reflect hysteresis in both types of mechanisms. Persistent contour integration could also involve sustained neural activity in more anterior cortical areas that play a top–down role in visual memory (Curtis and D’Esposito, 2003).

It is worth noting that our proposed feedforward-feedback account is not the only possible explanation for our results. An alternative account could predict greater persistence for smooth contours without the need for recurrent processing loops. For instance, contour onset could elicit transient activity in orientation-selective neurons, and during this initial surge of neural activity (which could occur within <100 ms), contour integration mechanisms (e.g., the association field) could enhance the representation of contour elements and their configuration, which could be transferred to a higher tier cortical area. Once transferred, it is possible that this high-tier representation no longer receives input from earlier visual areas (e.g., after the initial ∼100 ms surge of activity), and thus decays. While this is possible, it is not clear why this initial higher-tier representation should be stronger for smooth versus jagged contours since local orientation information is thought to be less important than global form in high-tier cortical representations of contour shape (Altmann et al., 2003; Kourtzi et al., 2003). Moreover, results from fMRI studies of contour persistence show an effect of familiarity on persistent neural activity in early visual areas, including V1 (Strother et al., 2011). Additionally, persistent neural activity in V1 was subsequently shown to be limited to the retinal location of the contour elements, and also to correspond to the duration of contour visibility (Strother et al., 2012). It should nevertheless be acknowledged that persistent neural activity in early visual areas, such as V1, could be epiphenomenal rather than evidence of a recurrent feedback loop between visual cortical areas. Further studies are necessary to test whether perceptual decay of a camouflage contour corresponds to persistent shape representation in high-tier visual cortical areas, earlier visual areas such as V1, or the persistent activation of a recurrent processing loop between several areas.

To conclude, the results reported here were obtained using a novel psychophysical method, and show that the neural mechanisms responsible for contour integration exhibit short-term memory, the duration of which is sensitive to the spatial properties of visual elements comprising the contour. Future studies could employ a more continuous range of element orientations and test for a possible within-observer correlation between contour detection performance and contour persistence. If observed, a correlation would strengthen the link between contour persistence and its neural basis in the association field. Additional studies could also employ neurophysiological measures to assess the concurrent operation of feedforward and feedback processes during persistent contour integration.

## Conflict of Interest Statement

The authors declare that the research was conducted in the absence of any commercial or financial relationships that could be construed as a potential conflict of interest.

## Footnotes

1 https://sites.google.com/site/visualformpersists/

2 https://sites.google.com/site/strothertoronto/

## SUPPLEMENTARY MATERIAL

Figure S1 **Gaze position results from a control experiments with three subjects (S1, S2, S3).** To obtain the results of **Figure 5B**, trials were excluded when gaze position deviated >1.5° from the fixation cross at the center of the screen. This means that the resultant data did not include trials for which the observer shifted his or her gaze to the actual contour (which occurred for <5% of trials for each observer). The smallest dashed circle indicates a distance of 1.5° from the fixation cross (distance used for filtering trials); the largest dashed circle indicates size of largest circle (4.5°); and the intermediate-sized dashed circle above indicates the size of the smallest contour circle used in the experiment.
