# Supplementary material for: Inter-element orientation and distance influence the duration of persistent contour integration
Source: Front Psychol. 2014 Nov 6;5:1273. doi: 10.3389/fpsyg.2014.01273 (PMC4222348; doi:10.3389/fpsyg.2014.01273)
Supplement: Figure S1 — Gaze position results from a control experiments with three subjects (S1, S2, S3). To obtain the results of Figure 5B, trials were excluded when gaze position deviated >1.5° from the fixation cross at the center of the screen. This means that the resultant data did not include trials for which the observer shifted his or her gaze to the actual contour (which occurred for <5% of trials for each observer). The smallest dashed circle indicates a distance of 1.5° from the fixation cross (distance used for filtering trials); the largest dashed circle indicates size of largest circle (4.5°); and the intermediate-sized dashed circle above indicates the size of the smallest contour circle used in the experiment. [file Data_Sheet_1.DOC]

**Supplementary Material**

(URL’s for stimuli were provided in the Introduction)


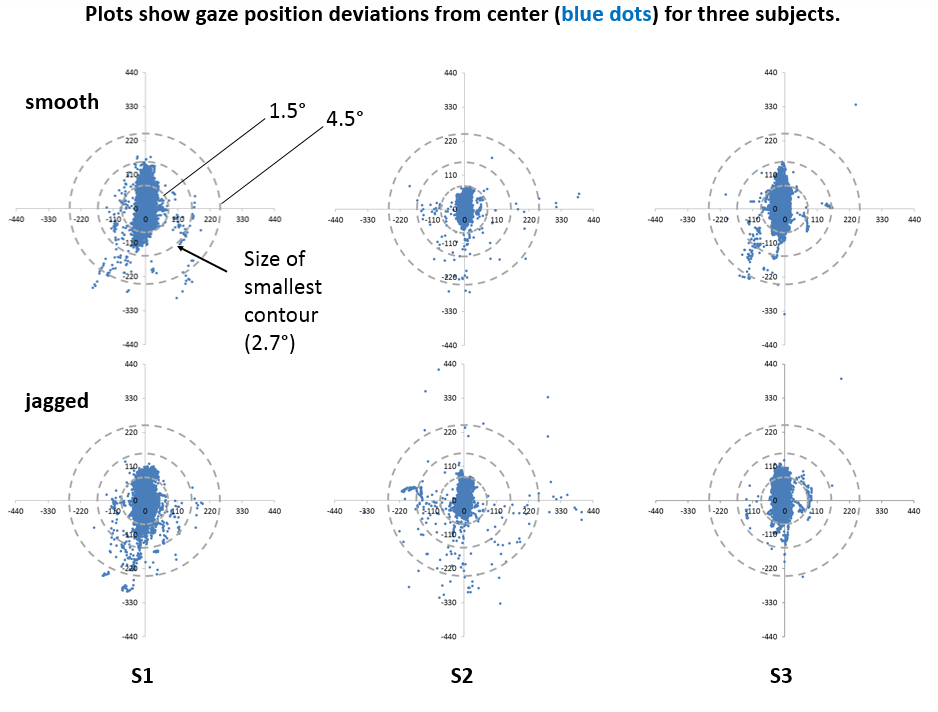


Figure S 1 Gaze position results from a control experiments with three subjects (S1, S2, S3). To obtain the results of Figure 5b, trials were excluded when gaze position deviated > 1.5° from the fixation cross at the center of the screen. This means that the resultant data did not include trials for which the observer shifted his or her gaze to the actual contour (which occurred for < 5% of trials for each observer). The smallest dashed circle indicates a distance of 1.5° from the fixation cross (distance used for filtering trials); the largest dashed circle indicates size of largest circle (4.5°); and the intermediate-sized dashed circle above indicates the size of the smallest contour circle used in the experiment.
